# Inferring human neutral genetic variation from craniodental phenotypes

**DOI:** 10.1093/pnasnexus/pgad217

**Published:** 2023-07-03

**Authors:** Hannes Rathmann, Silvia Perretti, Valentina Porcu, Tsunehiko Hanihara, G Richard Scott, Joel D Irish, Hugo Reyes-Centeno, Silvia Ghirotto, Katerina Harvati

**Affiliations:** Senckenberg Centre for Human Evolution and Palaeoenvironment, University of Tübingen, Tübingen 72070, Germany; Paleoanthropology, Institute for Archaeological Sciences, Department of Geosciences, University of Tübingen, Tübingen 72070, Germany; Department of Life Sciences and Biotechnology, University of Ferrara, Ferrara 44121, Italy; Department of Life Sciences and Biotechnology, University of Ferrara, Ferrara 44121, Italy; Department of Anatomy, Kitasato University School of Medicine, Sagamihara 252-0374, Japan; Department of Anthropology, University of Nevada, Reno, NV 89557, USA; Research Centre in Evolutionary Anthropology and Palaeoecology, School of Biological and Environmental Sciences, Liverpool John Moores University, Liverpool L3 3AF, UK; The Centre for the Exploration of the Deep Human Journey, University of the Witwatersrand, Johannesburg WITS 2050, South Africa; Department of Anthropology, University of Kentucky, Lexington, KY 40506, USA; William S. Webb Museum of Anthropology, University of Kentucky, Lexington, KY 40504, USA; DFG Center for Advanced Studies ‘Words, Bones, Genes, Tools’, University of Tübingen, Tübingen 72070, Germany; Department of Life Sciences and Biotechnology, University of Ferrara, Ferrara 44121, Italy; Senckenberg Centre for Human Evolution and Palaeoenvironment, University of Tübingen, Tübingen 72070, Germany; Paleoanthropology, Institute for Archaeological Sciences, Department of Geosciences, University of Tübingen, Tübingen 72070, Germany; DFG Center for Advanced Studies ‘Words, Bones, Genes, Tools’, University of Tübingen, Tübingen 72070, Germany

**Keywords:** cranium, dentition, neutral evolution, genetic variation

## Abstract

There is a growing consensus that global patterns of modern human cranial and dental variation are shaped largely by neutral evolutionary processes, suggesting that craniodental features can be used as reliable proxies for inferring population structure and history in bioarchaeological, forensic, and paleoanthropological contexts. However, there is disagreement on whether certain types of data preserve a neutral signature to a greater degree than others. Here, we address this unresolved question and systematically test the relative neutrality of four standard metric and nonmetric craniodental data types employing an extensive computational genotype–phenotype comparison across modern populations from around the world. Our computation draws on the largest existing data sets currently available, while accounting for geographically structured environmental variation, population sampling uncertainty, disparate numbers of phenotypic variables, and stochastic variation inherent to a neutral model of evolution. Our results reveal that the four data types differentially capture neutral genomic variation, with highest signals preserved in dental nonmetric and cranial metric data, followed by cranial nonmetric and dental metric data. Importantly, we demonstrate that combining all four data types together maximizes the neutral genetic signal compared with using them separately, even with a limited number of phenotypic variables. We hypothesize that this reflects a lower level of genetic integration through pleiotropy between, compared to within, the four data types, effectively forming four different modules associated with relatively independent sets of loci. Therefore, we recommend that future craniodental investigations adopt holistic combined data approaches, allowing for more robust inferences about underlying neutral genetic variation.

**Significance Statement**

Craniodental features are routinely used in bioarchaeology, forensics, and paleoanthropology to infer genetic relatedness across human remains. However, it is unclear whether certain data types preserve neutral evolutionary signals to a greater degree than others. Here, we test the relative utility of four standard metric and nonmetric data types, employing an extensive computational genotype–phenotype comparison across worldwide modern populations. Our results reveal that the four data types capture different amounts of neutral genomic variation, with dental nonmetrics and cranial metrics showing the highest signals and dental metrics displaying the lowest. Importantly, combining different data types maximizes genotypic coverage over different loci compared with using them separately. Therefore, we recommend prioritizing combined data sets for more accurate craniodental inferences in future research.

## Introduction

Human skeletal morphology is highly diverse and varies among individuals and populations across the globe. This pattern was shaped by the complex interplay of neutral evolutionary processes (i.e. selectively neutral mutations, random genetic drift, and gene flow) and nonneutral forces related to local adaptation and developmental plasticity in response to environmental and cultural stimuli (1–3). Different parts of the skeleton (such as the cranium, mandible, teeth, pelvis, long bones, hands, and feet) have been shown to preserve neutral and nonneutral signatures to different degrees (4–13). Overall, however, there is wide consensus that cranial and dental morphology as a whole evolved for a large part under neutrality and, thus, can be used as a proxy for reconstructing population structure and history (14–20). This is relevant for the study of human skeletal remains from archaeological and forensic contexts, where DNA analyses are often constrained due to poor molecular preservation, particularly in the deep fossil record and in warmer climates, or when destructive DNA sampling of fragile and rare specimens is not possible.

Morphological investigations based on craniodental features typically focus on either quantitative (hereafter, metric) or qualitative (hereafter, nonmetric) data to characterize the overall geometry of study specimens. Cranial metric data collection is performed by defining a set of homologous anatomical landmarks located on the skull and by measuring either linear dimensions between them (21, 22) or the relative position of landmark and semilandmark coordinates in two or three dimensions (23–26). Dental metric data collection is performed in a similar fashion, usually by measuring linear lengths, widths, or diagonal dimensions at the tooth crown or at the cement–enamel junction (27, 28) or semilandmark-based crown outlines (29, 30). Cranial nonmetric trait data collection is performed by visually scoring minor discontinuous variants, such as extra-sutural ossicles, proliferative ossifications including bridges or spurs, or variation in foramina number and location, for example (31–33). Similarly, dental nonmetric trait data collection is performed by observing the number of cusps and roots, or the pattern of fissures, ridges, and grooves on tooth crowns (34–37).

Despite the popularity of all four craniodental data types in population structure and history studies, it remains poorly understood whether some preserve neutral genomic signatures to a greater degree than others. This is problematic because investigations based on different craniodental data types may arrive at markedly disparate conclusions. For example, some researchers have suggested that teeth are a “safe box” of the genetic code, much more than any other skeletal element, because they form relatively early during ontogeny and their morphology remains unchanged after full formation, making teeth less affected by external stimuli (38). Some have also hypothesized that metric data are more useful than nonmetric traits, because measurements can be collected in a more objective and consistent manner, whereas visual scoring of nonmetric traits can be subjective and prone to observer error (39, 40). A vast body of literature also suggest varying levels of heritability among the different craniodental data types, with disparate amounts of genetic integration through pleiotropy, indicating that some types of data contain more independent genomic information than others (41–48). Several studies also point out that more holistic approaches combining different craniodental data types in a single analysis capture more phenotypic and thus genomic variation, compared with using them separately (49–51). Lastly, it has been proposed that there are not only differences in neutrality between the craniodental data types, but also differences within a given data type. That is, some bones, single trait expressions, or functional and developmental modules conserve a stronger evolutionary neutral signal than other, more labile, regions (10, 14, 17, 20).

A standard approach for quantifying the utility of a given craniodental data type in capturing a neutral genomic signature is to estimate phenotypic distances among worldwide modern human populations, on the one hand, and to compare them to neutral genomic distances estimated among the same or closely matched set of populations on the other (1, 2, 52). These analyses, hereafter termed *D*_P_–*D*_G_ comparisons, have been extensively performed for cranial metric data (14, 16, 17, 19, 20, 51, 53, 54), dental metric data (18, 55), cranial nonmetric trait data (51, 56, 57), and dental nonmetric trait data (10, 15, 18, 58). However, the estimated levels of neutrality of the different craniodental data types reported in previous *D*_P_–*D*_G_ studies are not directly comparable, since different populations have been sampled and diverse methodological approaches for calculating between-population distances have been employed at different geospatial scales (54).

To date, only few *D*_P_–*D*_G_ studies have attempted to systematically co-analyze the relative neutrality of different craniodental data types in a single analytical framework, thus, allowing for comparability (18, 51, 56). Those investigations found contradicting results, reporting either similar degrees of neutrality for different data types (18) or that they were differentially associated with genomic markers (51, 56). However, those previous studies were constrained by several factors. First, they were limited to either cranial (51, 56) or dental (18) data and none compared all four craniodental data types together. Second, none of the previous studies assessed the utility of a mixed-type data set combining metric and nonmetric traits in a single analysis. Third, none of these studies accounted for geographically structured environmental variation that can affect phenotypic and genomic variation (12). Fourth, all used rather limited sets of matched populations with varying and sometimes small sample sizes without accounting for variation introduced by sampling uncertainty. Fifth, all studies compared craniodental data types with unequal numbers of variables, which leads to biased results since phenotypic distances based on many variables are more robust than those based on only a few (10, 59). Sixth and finally, all previous studies compared phenotypic distances to a single point estimate of genetic distance, which takes all sampled genomic loci into consideration; instead, phenotypic distances should be compared with multiple equally plausible neutral genetic distances by randomly sampling genomic loci in order to account for stochastic variation inherent to a neutral model of evolution (10, 52, 60).

In this study, we address these research gaps by using a global *D*_P_–*D*_G_ framework in which we jointly investigate the relative neutrality of the four different craniodental data types, plus a mixed-type data set combining all four types of data together. Our extensive computations draw on the largest existing genomic and phenotypic databases currently available, while accounting for geographically structured environmental variation, population sampling uncertainty, disparate numbers of phenotypic variables, and stochastic variation inherent to a neutral model of evolution.

## Results

**Fig. 1.**
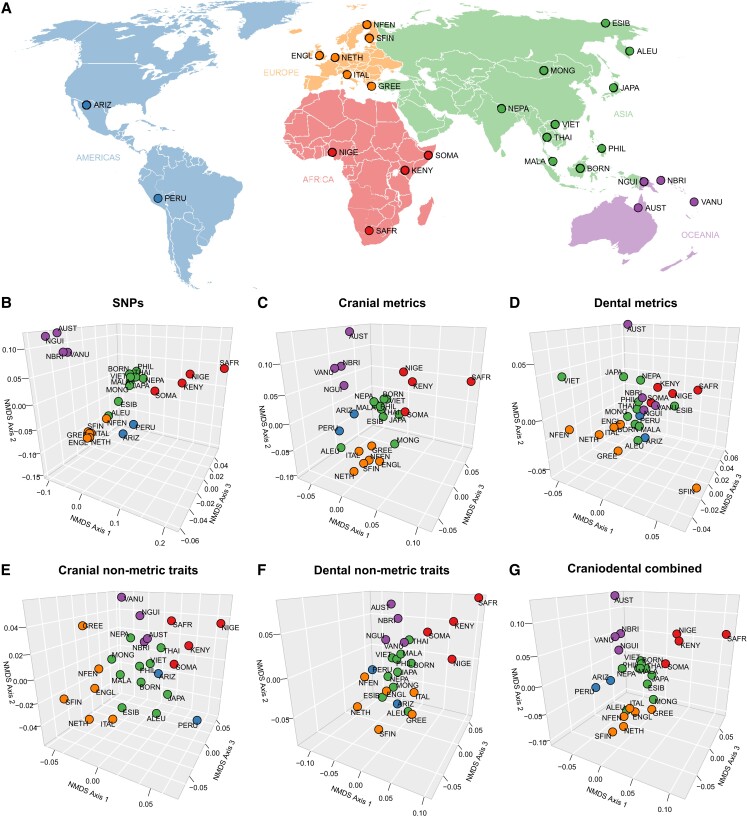
Geographic location and genomic and phenotypic relationships of worldwide modern human populations. (A) World map showing the locations of 26 populations sampled for matched genomic data (SNPs) and phenotypic data (cranial and dental metrics and nonmetric traits). Points are approximate geographic coordinates of the genomic samples. 3D NMDS plots of between-population distances, calculated separately from six different data types: (B) SNPs; (C) cranial metrics; (D) dental metrics; (E) cranial nonmetric traits; (F) dental nonmetric traits; and (G) combined craniodental data.

Mining large existing databases, we matched five different genomic and phenotypic data types for 26 modern human population samples from around the world, namely: (i) 8,821 single nucleotide polymorphisms (SNPs), (ii) 37 cranial metrics (in the form of linear dimensions, arcs, chords, and subtenses), (iii) 28 dental metrics (in the form of mesiodistal and buccolingual crown diameters), (iv) 24 cranial nonmetric traits, and (v) 25 dental nonmetric traits (Fig. 1A and SI Appendix, Table S1). We then estimated pairwise between-population genetic distances using Weir–Cockerham's *F_ST_* derived from the SNP data, which served as a benchmark to evaluate neutral expectations (Data Set S1). Next, we estimated pairwise between-population phenotypic distances using Mahalanobis’ *D*^2^, generated separately from the cranial metrics, dental metrics, cranial nonmetric traits, dental nonmetric traits, and the combined craniodental data (Data Sets S2–S6). We then subjected the *F_ST_* and *D*^2^ distances to Kruskal's nonmetric multidimensional scaling (NMDS) to visualize the matrices in a decomposed three-dimensional (3D) coordinate space, where a spatial grouping of populations indicates close affinity, and vice versa (Fig. 1B–G). The MDS stress level for the *F_ST_* matrix was 0.0407, and the stress levels for the cranial metric, dental metric, cranial nonmetric traits, dental nonmetric traits, and combined craniodental data *D*^2^ matrices were 0.0539, 0.1185, 0.1392, 0.0683, and 0.0615, respectively. All stress levels are below the acceptable threshold of 0.15, indicating that 3D captures the overall among-population variation well. To spatially orient the *D*^2^ distance configurations similar to the *F_ST_* distance configuration, we subjected the decomposed *D*^2^ coordinates to Procrustes superimposition to scale and rotate them to maximum similarity with the decomposed *F_ST_* coordinates by minimizing the overall sum of squared differences among populations. All 3D NMDS plots show major continental clusters of populations. The clusters appear most markedly geographically structured in the SNP data (Fig. 1B) and to a similar degree in the combined craniodental data (Fig. 1G), whereas clusters in the dental metric data appear least structured geographically (Fig. 1D).

**Fig. 2.**
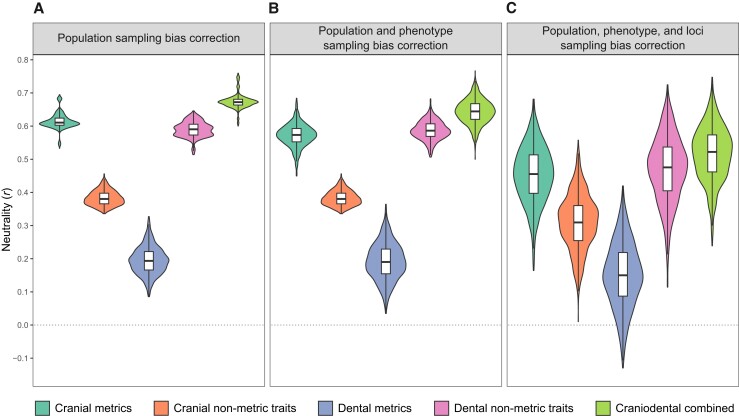
Violin plots showing neutrality estimates for five craniodental data types, calculated as partial Pearson correlation (*r*) between phenotypic (*D*^2^) and neutral genetic (*F_ST_*) distances across 26 modern human population samples, controlling for climate distances. Box plots are superimposed to show median values (black solid line) and interquartile ranges (boxes). (A) Distribution of 1,000 iteratively generated *r* values, each iteration leaving out a randomly selected population in the phenotypic and genomic data sets and a randomly selected individual in each remaining population. (B) Distribution of 1,000 iteratively generated *r* values, each iteration randomly undersampling the number of phenotypic variables, combined with population sampling bias correction. (C) Distribution of 1,000 iteratively generated *r* values, each iteration randomly undersampling the number of loci, combined with population and phenotype sampling bias correction.

To formally quantify the neutral signals preserved in a given phenotypic data type, we conducted partial correlation tests to measure the degree of congruence between *D*^2^ and *F_ST_*, while controlling for the effects of geographically structured environmental variation on phenotypic and genomic variation (12). Computationally, the partial correlation test design calculates the correlation of the residuals from the independent regressions *D*^2^ ∼ *C* and *F_ST_* ∼ *C*, whereby *C* describes climatic differences among sampled population environments (Data Set S7). The resulting partial correlation value *r* was treated as a neutrality estimate, with an *r* value close to 1 indicating a higher degree of neutrality, whereas an *r* value near to 0 indicates a lower degree. We obtained the highest *r* value for the combined craniodental data (*r* = 0.684), followed by cranial metrics (*r* = 0.618), dental nonmetric traits (*r* = 0.592), cranial nonmetric traits (*r* = 0.390), and dental metrics (*r* = 0.223). Similar patterns were observed when comparing *D*^2^ to *F_ST_*, while controlling for geographic distances (*G*) (Data set S8), albeit with slightly lower overall *r* values (SI Appendix, Table S2). However, due to variations in sample sizes between the matched phenotypic and genomic data sets (SI Appendix, Table S1), the *D*^2^ and *F_ST_* distances are statistically biased, and in consequence, the neutrality estimate *r*. Therefore, to explore the effect of population sampling uncertainty, we employed a resampling procedure whereby we calculated the neutrality estimator 1,000 times, each time leaving out a randomly selected population in the phenotypic and genomic data sets and a randomly selected individual in each remaining population. We then reported the median of the resulting distribution of *r* values and constructed an interpercentile range accounting for 95% of the spread. The results are summarized in Table 1 and visualized in Fig. 2A using violin plots. Overall, the highest distribution of *r* values was again attained for the combined craniodental data, followed by cranial metrics, dental nonmetric traits, cranial nonmetric traits, and dental metrics. To statistically corroborate this finding, we conducted repeated-measures *t*-tests among pairs of distributions (SI Appendix, Table S3) and found significant differences in the levels of neutral signals preserved in each craniodental data type (*P* < 0.001).

**Table 1.** Neutrality estimates for five craniodental data types, calculated as partial Pearson correlation (*r*) between phenotypic (*D*^2^) and neutral genetic (*F_ST_*) distances across 26 modern human population samples, controlling for climate distances.

Craniodental data type	Population sampling bias correction^a^	Population and phenotype sampling bias correction^b^	Population, phenotype, and loci sampling bias correction^c^
Cranial metrics	0.610 (0.553–0.682)	0.573 (0.498–0.640)	0.455 (0.276–0.610)
Cranial nonmetric traits	0.380 (0.346–0.430)	0.380 (0.346–0.430)	0.309 (0.138–0.464)
Dental metrics	0.194 (0.118–0.281)	0.190 (0.085–0.301)	0.150 (−0.043–0.338)
Dental nonmetric traits	0.591 (0.534–0.633)	0.586 (0.528–0.642)	0.476 (0.261–0.652)
Craniodental combined	0.672 (0.627–0.743)	0.644 (0.568–0.716)	0.522 (0.349–0.668)

a Median (and 95% range) of 1,000 iteratively generated *r* values, each iteration leaving out a randomly selected population in the phenotypic and genomic data sets and a randomly selected individual in each remaining population.

b Median (and 95% range) of 1,000 iteratively generated *r* values, each iteration randomly undersampling the number of phenotypic variables, combined with population sampling bias correction.

c Median (and 95% range) of 1,000 iteratively generated *r* values, each iteration randomly undersampling the number of loci, combined with population and phenotype sampling bias correction.

The five phenotypic data sets in our analysis comprise unequal numbers of variables (SI Appendix, Table S1), namely: 37 cranial metric variables; 24 cranial nonmetric trait variables; 28 dental metric variables; 25 dental nonmetric trait variables; and the combined craniodental data set comprises a summed up total of 114 variables. This imbalance hampers a direct comparison across data types, given that phenotypic analyses based on many variables are more robust than those based on only a few (10, 59). Therefore, to create equally sized numbers of variables across all five phenotypic data sets, we calculated the neutrality estimator *r* for a given phenotypic data type 1,000 times, each time randomly undersampling the number of variables down to 24. This corresponds to the number of variables in the cranial nonmetric trait data set, comprising the fewest variables among all data sets compared. Phenotypic variable sampling bias correction was performed together with population sampling bias correction, to explore the combined effect of these two analytical refinements. On average, the resulting distributions of *r* values for the five phenotypic data types exhibit a similar relative ordering to the population sampling bias corrected *r* values alone, with the only difference that dental nonmetric traits show a higher preservation of neutral genomic signatures compared with cranial metrics (Table 1, Fig. 2B). Pairwise repeated-measures *t*-tests (SI Appendix, Table S4) confirmed that the neutral signals preserved in each craniodental data type significantly differ from one another (*P* < 0.001).

Under a neutral model of evolution, the *F_ST_* distance matrix, used as a benchmark for our comparisons, is just one of multiple equally plausible neutral genetic outcomes produced by stochastic variation (10, 52, 60). To account for this stochasticity, we calculated *F_ST_* and thus the neutrality estimator *r* for a given phenotypic data type 1,000 times, each time randomly undersampling the number of SNP loci down to the same number of phenotypic variables, namely 24. This sampling strategy is consistent with population and quantitative genetics theory, where a heritable, additive, and selectively neutral phenotype is approximately as informative about population differentiation as a single neutral genomic locus, regardless of how many loci influence the phenotype (61, 62). Loci undersampling was performed in conjunction with population sampling bias correction and phenotypic variable sampling bias correction, to investigate the combined effect of these three analytical refinements. On average, the resulting distributions of *r* values exhibit a similar relative ordering to those correcting for population and phenotypic variable sampling bias combined (Table 1, Fig. 2C). Pairwise repeated-measures *t*-tests (SI Appendix, Table S5) showed that all neutral signals differ significantly (*P* < 0.001).

We note that overall our phenotypic data sets exhibit imbalanced distributions of sexes, with more males represented than females. This bias can be problematic as sexual dimorphism in shape, size, and trait expression may introduce variation unrelated to neutral genomic variation. Although we implemented data preprocessing steps to correct for sexual dimorphism (see Materials and Methods), we conducted two additional analyses focusing on males only. One analysis utilized size-corrected metric data (SI Appendix, Table S6), while the other did not apply size correction to the metric data (SI Appendix, Table S7). The results from the male subsets generally follow the same pattern as those of the complete data set, albeit with slightly wider *r* value intervals when controlling for population sampling uncertainty, which is expected given the overall smaller sample size.

Lastly, we acknowledge that our results may be affected by small sample sizes for certain data types, particularly dental metrics and SNPs, which were represented by only a few individuals in some populations (SI Appendix, Table S1). Although our analysis accounts for sampling bias (as described above), we conducted a more cautious analysis focusing on a subset of 16 out of the 26 populations with phenotypic and genomic sample sizes of *n* ≥ 10 (SI Appendix, Table S8). The obtained results show patterns that generally align with those observed in the full data set, although with slightly higher overall *r* values. The only distinction lies in higher *r* values observed for cranial metrics compared with dental nonmetric traits. Therefore, in order to reconcile the findings of the full 26-population data set and the 16-population subset, we consider dental nonmetric traits and cranial metrics equally suitable for tracking neutral signatures until further samples become available for study.

## Discussion

To our knowledge, this study is the first to systematically co-analyze the relative utility of four widely used standard craniodental phenotypic data types in capturing neutral genomic variation, namely (i) cranial metrics, (ii) dental metrics, (iii) cranial nonmetric traits, and (iv) dental nonmetric traits, plus (v) a mixed-type data set combining all four data types together. We performed a comprehensive *D*_P_–*D*_G_ comparison across 26 worldwide populations, drawing on the largest existing phenotypic and genomic data sets currently available, and incorporating a range of analytical refinements commonly neglected in previous *D*_P_–*D*_G_ studies. In doing so, we demonstrated the importance of accounting for sampling uncertainty and showed that *r* neutrality estimates can vary substantially based on the composition of population samples and numbers of specimens included, even with large data sets as employed here. This is, for example, markedly expressed by the dental metric data in the full 26-population data set, with a point estimate of *r* = 0.223, which widens to a 95% range of *r* = 0.118–0.281 when accounting for sampling bias. We further demonstrated the importance of accounting for unevenly sized numbers of phenotypic variables when comparing relative levels of neutrality across phenotypic data sets. Specifically, in the full 26-population data set, cranial metrics exhibited higher levels of neutrality compared with dental nonmetric traits when no correction was applied, but this pattern reversed when the number of phenotypic variables was equalized across data sets through random undersampling. This result is in agreement with previous research finding that the validity of cranial metric and dental nonmetric trait distances in reflecting neutral expectations is contingent upon the number of variables employed (10, 59). On a related note, our undersampling procedure also takes into account the practical limitations of working with skeletal remains, particularly in bioarchaeological or fossil contexts, where craniodental data are often highly fragmented, and where researchers must work with random subsets of variables. Lastly, we demonstrated the importance of accounting for stochastic variation inherent to a neutral model of evolution by randomly undersampling the SNP loci to match the number of phenotypic variables. This resulted in *r* neutrality estimate distributions with much wider ranges, and for the dental metric data, the 95% range was found to be *r* = −0.043–0.338 (in the full 26-population data set) and *r* = 0.067–0.612 (in the 16-population subset), with the lower bounds near zero implying nonneutral evolutionary forces. This finding therefore calls into question the validity of dental metrics as a proxy for neutral genomic markers.

Inspecting the four craniodental data types separately, our results clearly show that they are differentially associated with neutral genomic variation after accounting for population sampling uncertainty, disparate numbers of phenotypic variables, and stochastic variation inherent to a neutral model of evolution. In testing for neutrality, our estimates reveal that, overall, dental nonmetric traits and cranial metrics performed best, followed at some distance by cranial nonmetric traits, whereas dental metrics performed relatively poorly. Interestingly, these estimates do not relate to the suggested general divide in utility between cranial versus dental features, with the latter proposed to be less affected by external environmental stimuli (38), and nonmetric versus metric data, with the latter suggested to be less prone to observer error (39). Instead, our estimates are in agreement with previous quantitative genetic studies of pleiotropy in humans (or in nonhuman primates when studies on humans are not yet available), finding that the amount of independent genetic information in dental metrics (41) and cranial nonmetric traits (45) is low, compared with the amount of independent genetic information in cranial metrics (44) and dental nonmetric traits (42, 43). The relatively poor performance of dental metrics contrasts with what was proposed in a previous study using a methodological *D*_P_–*D*_G_ framework similar to ours (18), which found that dental metrics and nonmetric traits are both comparably well-suited in tracking neutral genomic variation. The present study expands and improves upon the *D*_P_–*D*_G_ investigation by Rathmann et al. (18) in several respects. Among the most important are a more comprehensive dental nonmetric trait data set for comparison (25 versus 15 traits) and a larger set of globally distributed matched population samples (26 versus 19 populations).

Perhaps one of the most interesting findings of our study is that phenotypic inferences of neutral genomic variation are most accurate when based on a combined mixed-type data set, compared with using the four different data types separately. This result is in agreement with previous studies reporting that phenotypic inferences about genomic affinities are best served when multiple lines of evidence are jointly investigated (49–51). This is also expected, given that the number of variables in the mixed-type data set is many times higher than in the four different data sets separately, leading to a richer knowledge of phenotypic and thus genomic variation (10, 59). Interestingly though, when equalizing the numbers of phenotypic variables across all data sets via undersampling, the mixed-type data still performed best. One possible explanation for this result could be that genetic integration through pleiotropy between the four data types is lower than genetic integration within the four data types, effectively forming four different modules regulated by different sets of loci that are relatively independent from each other (63–65). In this situation, even when just a few phenotypic variables per data type would contribute to the mixed-type data, more underlying genomic variation from different loci would still be captured than using the full phenotypic variable battery of one of the four data types individually. This hypothesis could be tested with a quantitative genetic analysis of pleiotropy in a modern human population with known pedigree structure sampled for all four cranial and dental metric and nonmetric trait data types, which to our knowledge has not been performed so far and could thus lead to exciting new research directions.

We note that the reported *r* neutrality estimates for the different craniodental data types must be considered minimum values as they are biased toward zero. This is because we compared matched but unpaired population samples, with phenotypic and genomic data coming from different individuals; however, phenotypic and genomic distances among unpaired samples have a reduced degree of congruence, given that within-population variation is high compared with between-population variation (66). Nevertheless, comparing unpaired samples is a standard procedure in global scale *D*_P_–*D*_G_ analyses (7, 10, 14–20, 51, 55), and our applied analytical correction for sampling bias (i.e. both population and specimen resampling of the phenotypic and genomic data) may account for at least some of this uncertainty. Moreover, although our large craniodental data sets comprise the most widely used metric and nonmetric trait variables in bioanthropological research, they could be complemented with additional standard and nonstandard variables proposed to be informative (67–70). Similarly, the metric portion of our data sets, consisting of linear dimensions, arcs, cords, and subtenses, could be replaced with 3D coordinate data that better retain the geometry of the studied specimens than caliper-based measurements. Interestingly, though not fully comparable, previous *D*_P_–*D*_G_ analyses based on craniodental 3D data reported neutrality levels similar to those reported here (14, 17, 19, 20, 55), suggesting that caliper-based measurements and 3D coordinates are equally well-suited for reconstructing genetic relationships, though our caliper-based data sets have the advantage to be many times larger.

Previous studies proposed that there are not only differences in neutrality between the four craniodental data types, but also differences among the variables within a given data type (10, 14, 17, 20). Our phenotypic variable undersampling procedure takes at least some of these considerations into account and we show that neutrality estimates for a given data type differ substantially when different subsets of variables are employed, further reinforcing previous claims. Future investigations should therefore explore additional arrangements of variables beyond the five tested here. For instance, considering only cranial data, combining all nonmetric variables, utilizing variables with the highest discriminatory power, or focusing on variables associated with previously identified functional and developmental modules (9, 17, 48). We propose that testing for neutrality in all possible combinations of cranial and dental metric and nonmetric variables, as recently employed for dental nonmetric trait data (10), is the most promising approach, rather than restricting analysis to predefined or hypothesized arrangements only.

##  

In conclusion, our results serve as a reference for future craniodental research, confirming that most of the traditionally used data types can be used as proxies for neutral genomic data, although some are more useful than others. We do advise, however, to carefully review the use of dental metrics in the form of standard mesiodistal and buccolingual crown dimensions only, as they may not cover sufficient independent genomic variation, at least in comparison with other craniodental data types. Importantly, instead of using the different data types separately, we advise relying on a more holistic approach by combining them together, as this maximizes genotypic coverage over different loci resulting from primarily neutral evolution. Future work in combinatorics should focus on identifying specific subsets of mixed cranial and dental metric and nonmetric traits that are the most useful for tracking human neutral genetic variation.

## Materials and methods

### Matching population samples

Materials for this study comprise five different types of data: (i) SNPs, (ii) cranial metrics, (iii) dental metrics, (iv) cranial nonmetric traits, and (v) dental nonmetric traits. All data were taken from existing databases. We matched the different data types for 26 globally distributed modern human populations for which all five types of data were available (Fig. 1 and SI Appendix, Table S1). Populations were chosen for inclusion in this study based on three criteria: (i) availability of *n* ≥ 3 unrelated individuals per genetic sample; (ii) availability of *n* ≥ 4 individuals per phenotypic sample; and (iii) sample antiquity <2,000 years, to control for temporal bias. In instances where exact population matches between genotypic and phenotypic populations could not be achieved, a geographically proximate population with ethno-linguistic affinities was selected. In a few cases, closely related populations were pooled to maximize sample size. We note that the matched population samples in this study are unpaired; that is, all five types of data derive from different individuals. When possible, approximately equal numbers of adult males and females (determined osteologically) were sampled for the phenotypic data sets, to control for sexual dimorphism; however, we note that overall the phenotypic data sets are biased toward representing more males.

### SNP data

SNP data were obtained from published databases, genotyped with the Affymetrix Human Origins Array (71–80). To correctly merge genotypes coming from different data sets, we ensured they were all related to the same Reference Sequence, the Genome Reference Consortium Human Build 37 (81) using, when needed, the LiftOver tool (82). To merge data from selected data sets, we used the plink-1.90 software (83). We filtered the data removing all transversions to avoid ambiguity in strand alignment (C/G or A/T), principal component analysis outliers, and first- and second-degree relative pairs. We selected only those SNPs that map to nonfunctional genomic regions and are therefore unlikely to be affected by natural selection. We applied two different filter levels for the amount of allowed missing data: first, to populations collected by Lazaridis et al. (74), Mallick et al. (76), Pickrell and Pritchard (78), and Skoglund et al. (80), we retained only individuals with 0% missing data; second, from the other published resources, we removed individuals with >10% of missing data. All filtering was performed using the plink-1.90 software (83). Finally, we converted the data set from PLINK file format into a genepop file using PGDSpider (84). The final preprocessed SNP data set comprised 857 individuals sharing 8,821 markers, with population sample representation varying from 3 to 176 individuals.

### Cranial metric data

The cranial metric data were selected from a larger database collected by one of us (T.H.) (85). The data set consists of 37 measurements of the cranium recorded for each individual, in the form of linear dimensions, arcs, cords, and subtenses. All measurements were recorded following the procedures in Bräuer (70) using sliding and spreading calipers. Raw measurements were converted into scale-free shape variables by dividing each measurement by the geometric mean for all the measurements in each individual (86). This procedure removes gross size from the data in order to assess differences in the proportionate contribution of single variables to overall cranial size and adjusts for size differences between individuals that may result from sexual dimorphism. Because size-correction requires complete cases, missing values were imputed with the *k*-nearest neighbor (*k*NN) method (87). *k*NN searches the entire data set for *k* = 5 individuals most similar to the one with missing data and generates a mean to replace the missing value(s). Prior to imputation, individuals with more than half of the measurements missing were removed from the analysis. In this way, we ensured that <2.5% of the final data set requires imputation (down from 3.1%). Summary statistics of the *k*NN-imputed and size-corrected cranial metric data set are provided in Data Set S9. The final preprocessed cranial metric data set comprised 2,994 individuals, with population sample representation varying from 24 to 366 individuals.

### Dental metric data

The dental metric data were selected from a larger database collected by one of us (T.H.) (88). The data set consists of mesiodistal and buccolingual crown diameters of all teeth recorded for each individual (up to a total of 28 metric variables, excluding third molars). Only right teeth were measured, but when a right tooth was missing, damaged, or affected by wear or pathology, the corresponding left antimere was measured. All measurements were recorded according to the procedures in Moorrees (89) and Hillson (90) using a digital sliding caliper accurate to 0.01 mm. We applied the same data preprocessing steps as for the cranial metric data. First, individuals missing more than half of the measurements were removed to ensure that <24.3% of the data set requires imputation (down from 57.7%). Second, missing values were imputed using the *k*NN algorithm (87). Third, raw measurements were converted into scale-free shape variables (86) to assess differences in the proportionate contribution of individual variables to overall tooth size and to adjust for size differences that may result from sexual dimorphism (40). Summary statistics of the *k*NN-imputed and size-corrected dental metric data set are reported in Data Set S10. The final preprocessed dental metric data set comprised 909 individuals, with population sample representation varying from 4 to 185 individuals.

### Cranial nonmetric trait data

The cranial nonmetric trait data were selected from a larger database collected for the most part by one of us (T.H.) (91). The data set consists of 24 discrete observations of the cranium recorded for each individual and comprises data on sutural variation, supernumerary ossicles, hypostotic and hyperostotic traits, and vessel/nerve-related traits. The scoring procedures for each trait are described elsewhere [Hanihara et al. (91) and references therein]. Scoring followed the individual count method (92), where bilateral traits were counted only once per cranium, regardless of whether or not the trait appeared bilaterally. In cases where a trait was expressed asymmetrically, the side with the highest expression level was scored. Graded trait expression scores were collapsed into simplified binary dichotomies of absence or presence based on established breakpoints [Hanihara et al. (91) and references therein]. Sex differences were found in a few traits but none of the traits differed consistently between males and females in all sampled populations and we thus analyzed both sexes together, as it has been done in previous analyses of the same data set (91). Summary statistics of the cranial nonmetric trait data set are provided in Data Set S11. The final preprocessed cranial nonmetric trait data set comprised 4,623 individuals, with population sample representation varying from 26 to 533 individuals.

### Dental nonmetric trait data

The dental nonmetric trait data were obtained from published resources (15, 68), whereby the majority of the samples were collected by C. G. Turner II, later augmented with samples collected by two of us (G.R.S. and J.D.I.; SI Appendix, supplementary text 1). The data set consists of 25 discrete observations of the dentition, including data on the number of cusps and roots, and the pattern of fissures, ridges, and grooves on tooth crowns. All data collectors used the Arizona State University Dental Anthropology System (ASUDAS) to record trait observations (68, 93). The ASUDAS comprises a reference set of dental casts illustrating expression levels for various traits alongside specific instructions that ensure a standardized scoring procedure, which minimizes observer error. ASUDAS traits are routinely collected on key teeth (usually the most mesial member of a tooth district) because these are considered the most stable members in terms of development and evolution (94). As in the cranial nonmetric trait data set, scoring followed the individual count method (92). Dental trait expression scores were collapsed into simplified binary dichotomies of absence or presence based on established breakpoints (15, 68). Dental traits of the ASUDAS have little or no sexual dimorphism, thus, it is a standard procedure to pool sexes (42, 46, 68, 94). Summary statistics of the dental nonmetric trait data set are provided in Data Set S12. The dental nonmetric trait data set comprised 2,986 individuals, with population sample representation varying from 28 to 450 individuals.

### Estimating distances among populations

Pairwise neutral genetic distances among populations were computed from the SNP data using *F_ST_*, defined as the fixation (*F*) index comparing the subset (_*S*_) genetic diversity within populations to the total (_*T*_) genetic diversity of all sampled populations. We followed Weir and Cockerham’s method of moments for diploid loci and calculated *F_ST_* for each SNP individually, averaging *F_ST_* over all loci (95). Under this model, populations of the same size are considered to have descended from a common ancestral population, which is assumed to be in Hardy–Weinberg equilibrium (Data Set S1).

Pairwise phenotypic distances were calculated from the craniodental data using Mahalanobis’ D^2^ distance, a model-free measure accounting for correlation among variables to avoid over-representing variation from variables that co-occur. The *D*^2^ distance between two populations *i* and *j* is estimated as the difference between two vectors of variable averages (*X_i_* and *X_j_*), adjusted by a pooled within-population variance–covariance matrix (*S*) estimated over all populations in the analysis. For the cranial and dental metrics, we estimated *D*^2^ following Mahalanobis (96), where *X_i_* and *X_j_* are calculated as geometric means, and *S* is calculated as a pooled Pearson variance–covariance matrix weighted by population sample sizes (Data Sets S2 and S3). For the cranial and dental nonmetric traits, we estimated *D*^2^ following Nikita (97), where *X_i_* and *X_j_* are calculated as probit threshold values of trait frequencies, and *S* is calculated as a pooled Pearson correlation matrix weighted by the sample sizes for each pair of traits (Data Sets S4 and S5). When estimating *D*^2^ for the combined craniodental data, we first computed *D*^2^ independently for each of the four data types, and then combined the four *D*^2^ matrices as a weighted average based on the numbers of variables (Data Set S6). Although this approach is valuable for handling unpaired samples and accounts for correlations within the four data sets, it does not account for correlations between them. However, in our case, it may still be appropriate since previous research demonstrated that the different data types are largely independent from each other, at least when comparing cranial metrics, dental metrics, and dental nonmetric traits (27), or cranial nonmetric and dental nonmetric traits (98). In addition to model-free *D*^2^ distances, we also calculated model-bound *P_ST_* distances, which incorporate relative estimates of effective population size (*N*_e_; SI Appendix, Table S9) and average estimates of heritability (*h*^2^; SI Appendix, supplementary text 2). Results obtained with *P_ST_* show similarities to those using *D*^2^ (SI Appendix, Table S10). However, due to the challenge of validating the parameter estimates *N*_e_ and *h*^2^, we opted to rely on *D*^2^ in order to limit potential model bias.

Pairwise climatic distances among sampled population environments (*C*) were calculated as Euclidean distances based on five temperature-related variables obtained from a global climate database published in Hubbe et al. (9), using latitudes and longitudes approximated for each population sample (Data Set S7). As climate indicators for each population region, we used estimates of annual minimum temperature, annual maximum temperature, annual average temperature, maximum temperature of the warmest month, and minimum temperature of the coldest month, all measured in °C. These indicators are listed for each population sample in Data Set S13.

Pairwise geographic distances (*G*) were calculated as geodesic distances between population latitudes and longitudes (Data Set S8).

### Correlation tests

We conducted Pearson correlation tests between the off-diagonal values in any two distance matrices to measure the linear association between phenotypic (*D*^2^), genetic (*F_ST_*), climate (*C*), and geographic (*G*) distances. We used partial Pearson correlation tests based on the residuals of a previous correlation and the off-diagonal values in a third matrix to evaluate the linear association between *D*^2^ and *F_ST_*, while controlling for either *C* or *G*. The resulting *r* coefficients are reported in SI Appendix, Table S2. To account for population sampling uncertainty in our partial correlation tests of *D*^2^, *F_ST_*, and *C*, we calculated the *r* coefficients 1,000 times, each time leaving out a randomly selected population in the phenotypic and genomic data sets and a randomly selected individual in each remaining population. Additionally, to create equally sized numbers of variables across all phenotypic data sets, in each of the 1,000 iterations we randomly undersampled the number of variables down to 24, which corresponds to the number of variables in the cranial nonmetric trait data set, comprising the fewest variables among all phenotypic data sets being compared. Further, to account for stochastic variation inherent to a neutral model of evolution, in each of the 1,000 iterations we randomly undersampled the number of SNP loci down to the same number as there are phenotypic variables, namely, 24. To gauge the relative neutrality of the different phenotypic data types in a visual manner, we plotted the distributions of estimated *r* coefficients using violin plots. Statistical significance between pairs of distributions was evaluated with repeated-measures *t*-tests with the application of a Bonferroni correction for multiple testing (SI Appendix, Tables S3–S5).

Unless otherwise noted, all analyses were performed in R, version 4.2.2 (99). The data and R code are publicly accessible from the Zenodo repository at https://doi.org/10.5281/zenodo.8067443. World map in Fig. 1 modified from https://commons.wikimedia.org/wiki/File:BlankMap-World6.svg (Public Domain).

## Supplementary Material

pgad217_Supplementary_Data

## Data Availability

The data and code used for analyses are publicly accessible from the Zenodo repository at https://doi.org/10.5281/zenodo.8067443.
