# Supplementary material for: Inferring human neutral genetic variation from craniodental phenotypes
Source: PNAS Nexus. 2023 Jul 3;2(7):pgad217. doi: 10.1093/pnasnexus/pgad217 (PMC10338903; doi:10.1093/pnasnexus/pgad217)
Supplement: pgad217_Supplementary_Data [file pgad217_supplementary_data.zip › PNASNEXUS-PNASNEXUS-2023-00352R-s01.pdf]

Supporting Information for
Inferring human neutral genetic variation from craniodental phenotypes

Hannes Rathmann, Silvia Perretti, Valentina Porcu, Tsunehiko Hanihara, G. Richard Scott, Joel D. Irish, Hugo Reyes-Centeno, Silvia Ghirotto, Katerina Harvati

Corresponding author: Hannes Rathmann
Email: hannes.rathmann@uni-tuebingen.de

This PDF file includes:

Supplementary text 1
Supplementary text 2
Tables S1 to S10
Legends for Datafiles S1 to S13
SI References

Other supplementary materials for this manuscript include the following:

Datasets S1 to S13

Supplementary Text 1

Unlike the cranial metric, dental metric, and cranial non-metric trait datasets used in our global D_P–D_G framework, our dental non-metric trait dataset does not consist of individual-level observations. Instead, it summarizes trait frequencies per population sample. The individual-level observation data are only partially available to our disposal. This poses a challenge in computing D^2 distances, which require individual-level observations for calculating the pooled within-population Pearson correlation matrix S (see Materials and Methods). As dental non-metric traits recorded on key teeth are largely independent from each other (1, 2), S can be approximated with an identity matrix, where diagonal elements are set to 1 and off-diagonal elements to 0, reducing D^2 to an Euclidean distance, an approach widely followed in previous studies (3, 4). However, as this approach may oversimplify the true covariance structure of dental non-metric traits, we took a different approach and aimed at directly estimating S with individual-level data available to us. Specifically, we approximated S using individual-level data from a subset of 14 out of the 26 population samples used in our global D_P–D_G analysis. This subset included a total of 1,110 individuals out of the 3,470 individuals sampled for dental non-metric traits. The subset consisted of the following populations: PHIL (n=84), ESIB (n=125), JAPA (n=131), BORN (n=40), VIET (n=26), MALA (n=57), GREE (n=70), ITAL (n=55), SOMA (n=76), KENY (n=60), SAFR (n=212), NIGE (n=28), ALEU (n=95), and SFIN (n=51). The S correlation matrix estimated from this data subset is provided in Dataset S12. In broad agreement with the standard assumption in dental morphological research, our correlation results indicate that dental non-metric traits are weakly correlated with each other, as all pairwise correlations range between -0.144 and 0.281. We consider our estimated correlation matrix as a suitable proxy for the true covariance structure of the entire dental non-metric trait dataset used in our global D_P–D_G framework, and a superior solution to a simple identity matrix.

When accounting for population sampling uncertainty in our analysis (see Materials and Methods), we approximated the effect of leaving out a randomly selected individual in our dental non-metric trait dataset via a simulation approach. Specifically, we created an artificial individual-level dental non-metric trait dataset consisting of randomly generated absence (i.e., 0) and presence (i.e., 1) and missing data (i.e., NA) scores, based on the information available for each population sample, namely, 1) number of scorable observations per trait, 2) number of negative observations per trait, 3) number of positive observations per trait, and 4) overall number of individuals observed in the sample. The artificial individual-level dataset generated in this manner results in population-level trait frequencies identical to the empirically observed data. This approach proved valuable for approximating the variation introduced by resampling individuals.

While metric dimensions of the cranium and dentition often exhibit significant sexual dimorphism, with males typically slightly larger than females, dental non-metric traits recorded on key teeth generally do not display such dimorphism, an observation made for thousands of dentitions studied in different samples across the globe (1, 2, 5, 6). The only trait identified to date that may show some levels of sexual dimorphism consistently across diverse samples is the distal accessory ridge on upper and lower canines, which appears slightly more pronounced in males compared to females when expressed (1, 2). However, this trait is not included in our database. Therefore, we consider the effect of sexual dimorphism on our dental non-metric trait dataset to be negligible. Consequently, when generating our artificial individual-level dataset, we randomly assigned sexes (male or female) to individuals at an approximate ratio of 2:3, aligning with the overall sex ratio observed in our other datasets for cranial metric, dental metric, and cranial non-metric traits.

Supplementary Text 2

The pairwise phenotypic distance measure D^2 utilized in our global D_P–D_G framework can be regarded as "model-free" (7, 8) in the sense that it describes statistical shape differences among sampled populations without relying on a specific theoretical population genetics model. An alternative approach is to employ "model-bound" phenotypic inferences based on P_{ST}, a measure of phenotypic population differentiation considered analogous to F_{ST} (9–11). P_{ST} is closely related to D^2 but can be scaled with an average estimate of heritability (h^2) and weighted with estimates of effective population size per sample (N_e), the latter being useful to account for the confounding effects of genetic drift on small populations. To allow comparisons with P_{ST}–based D_P–D_G studies, we reanalyzed our data with the respective P_{ST} method. P_{ST} values were calculated following the R-matrix method (12, 13), employing the D^2-to-P_{ST} conversion procedures outlined in ref. (7).

In our calculations of P_{ST}, we followed the approach outlined in ref. (9) and assumed full heritability (h^2=1), without distinguishing differential h^2 values for the different craniodental metric and non-metric data types. Although some heritability estimates for craniodental variables are available, they are population-specific and vary widely even for the same populations (5, 6, 14–20). We note that while the value of h^2 affects the magnitude of P_{ST}, the change in the value is proportional pairwise and would therefore not affect subsequent matrix correlation analyses with F_{ST}.

We included point estimates of effective population size (N_e) derived from our SNP data, calculated from levels of linkage disequilibrium following the procedures outlined in ref. (21), reported in Table S9. It is important to note that several of the obtained N_e estimates are based on small sample sizes and exhibit wide confidence intervals, making them less reliable. This is particularly evident for the NFEN sample (n=3), where the upper confidence interval for N_e extends to infinity. Similarly, the calculated N_e value for the African KENY sample (n=4) appears to be low and falls outside the range of African N_e values reported in this and previous studies (22). Therefore, caution must be exercised when interpreting our P_{ST} measures, and we opted to rely on D^2 in order to limit potential model bias. Nonetheless, the overall D_P–D_G results using P_{ST}, as reported in Table S10, are broadly similar to those using D^2. Previous studies have also shown a similar level of correlation between F_{ST} and D^2, and between F_{ST} and P_{ST}, even when N_e is included (9), suggesting that "model-free" D^2 distances serve as a suitable proxy for "model-bound" P_{ST} distances.

Table S1. Matched phenotypic and genetic population samples, geographic coordinates, numbers of variables, and sample sizes.

Population (Abbrev.)	Lat	Long	Cranial metrics (37 variables)		Dental metrics (28 variables)		Cranial non-metric traits (24 variables)		Dental non-metric traits (25 variables)		SNPs (8821 variables)	
			n (males / females / unknown)	sample names and reference	n (males / females / unknown)	sample names and reference	n (males / females / unknown)	sample names and reference	n (males / females / unknown)	sample names and reference	n (total)	sample names and reference
Aleutian Islands (ALEU)	53.6	160.8	285 (160/125/0)	Aleut1, Aleut2, Aleut3, Aleut4, Aleut5 [1]	47 (35/12/0)	Aleut [2]	370 (199/160/11)	Aleut1, Aleut2, Aleut3, Aleut4, Aleut5 [3]	95 (63/32/0)	Aleuts (Western US) [4]	7	Aleut [6]
Arizona (ARIZ)	29	-108	76 (41/35/0)	Arizona [1]	15 (11/4/0)	Arizona [2]	103 (61/42/0)	Arizona [3]	165 (110/55/0)	Pima 94 [4]	14	Pima [6]
Australia (AUST)	-13	143	35 (33/2/0)	Australia NT, Australia Queensland [1]	5 (3/2/0)	Australia NT, Australia Queensland [2]	64 (29/33/2)	Australia NT, Australia Queensland [3]	57 (38/19/0)	Australia—North BP [4]	10	Australian_ECCAC, Australian_WGA [6,7]
Borneo (BORN)	0	115	106 (96/10/0)	Borneo 1, Borneo 2 [1]	32 (27/5/0)	Borneo [2]	151 (112/39/0)	Borneo [3]	144 (96/48/0)	Borneo 94 [4]	17	Borneo, Lebbo [7,8]
Eastern Siberia (ESIB)	69.5	168.8	25 (21/4/0)	Chukchi [1]	4 (4/0/0)	Chukchi [2]	30 (21/5/4)	Chukchi [3]	126 (84/42/0)	Chukchi plus Eastern Siberia [4]	23	Chukchi, Chukchi_Reindeer, Chukchi_Sir [6]
England (ENGL)	52.68	-3.15	346 (239/107/0)	Pundbury, Spitalfields CAM, Spitalfields NHM [1]	185 (140/45/0)	Pundbury, Spitalfields CAM, Spitalfields NHM [2]	553 (321/200/32)	Pundbury, Spitalfields CAM, Spitalfields NHM [3]	131 (87/44/0)	English [5]	24	United_Kingdom_POPRES [9]
Greece (GREE)	38	23.7	130 (65/65/0)	Greek recent [1]	15 (14/1/0)	Greek [2]	132 (60/72/0)	Greek recent [3]	70 (47/23/0)	Greek Recent [4]	43	Greek_Coriell, Greek_Comas, Greek_WGA, Greece_POPRES [6,9]
Italy (ITAL)	43	11	99 (99/0/0)	Italy recent [1]	34 (34/0/0)	Italy recent [2]	146 (104/33/9)	Italy recent [3]	55 (37/18/0)	Italy Modern [4]	33	Italian_Bergamo, Italian_EastSicilian, Italian_South, Italian_Tuscan, Italian_WestSicilian, Italian_Tuscan [6,10]
Japan (JAPA)	38	138	218 (150/68/0)	Japan Tohoku, Japan Tokyo [1]	54 (50/4/0)	Japan [2]	189 (121/68/0)	Japan [3]	131 (87/44/0)	Recent Japanese [4]	29	Japanese [6]
Kenya (KENY)	-0.4	36.9	121 (92/29/0)	Kenya [1]	31 (26/5/0)	Kenya [2]	196 (102/90/4)	Kenya [3]	60 (40/20/0)	Kikuyu [4]	4	Kikuyu [6]
Malaysia (MALA)	4.2	102	39 (36/3/0)	Malay [1]	19 (18/1/0)	Malay [2]	40 (37/3/0)	Malay [3]	58 (39/19/0)	Malay Composite [4]	9	Malays [8]
Mongolia (MONG)	45	111	212 (148/64/0)	Mongol [1]	78 (46/32/0)	Mongol [2]	281 (163/115/3)	Mongol [3]	82 (55/27/0)	Mongol 2 and 3 Pooled [4]	10	Mongolians, Mongolians [6,10]
Nepal (NEPA)	28.1	82.5	42 (35/7/0)	India-Nepal [1]	10 (10/0/0)	India-Nepal [2]	53 (37/13/3)	India-Nepal [3]	97 (65/32/0)	Nepal 94 BP [4]	10	Kusunda [6]
Netherlands (NETH)	52.37	4.87	37 (35/2/0)	Holland [1]	11 (11/0/0)	Holland [2]	44 (35/9/0)	Holland [3]	72 (48/24/0)	Netherlands [5]	16	Netherland_POPRES [9]
New Britain (NBRI)	-5.8	150.8	95 (71/24/0)	New Britain	66 (50/16/0)	New Britain [2]	268 (144/118/6)	New Britain [3]	238 (159/79/0)	New Britain 1_4 738 BP_ no 3 [4]	156	New Britain [8]
New Guinea (NGUI)	-4	143	152 (97/55/0)	Papua New Guinea Central Prov, Papua New Guinea Eastern Islands, Papua New Guinea Gulf Prov, Papua New Guinea Madang Prov,Papua New Guinea Milne Bay Prov [1]	31 (20/11/0)	Papua New Guinea, Papua New Guinea 1, Papua New Guinea 2, Papua New Guinea 3 [2]	513 (239/260/14)	Papua New Guinea [3]	350 (233/117/0)	New Guinea and New Guinea Gulf [5]	49	Papuan, New_Guinea, Papuan [6,7,11]
Nigeria (NIGE)	7.4	3.9	102 (78/24/0)	Nigerai Ibo [1]	61 (50/11/0)	Nigerai Ibo [2]	164 (83/76/5)	Nigerai Ibo [3]	28 (19/9/0)	Yoruba [4]	85	Igbo, Yoruba [6,12]
Northern Fenno-scandia (NFEN)	68.4	23.6	37 (34/3/0)	Lapps [1]	6 (5/1/0)	Lapps [2]	42 (34/8/0)	Lapps [3]	64 (43/21/0)	Lapps (Kola Peninsula) [4]	3	Saami_WGA, Saami [6,13]
Peru (PERU)	-13.5	-72	366 (223/143/0)	Peru [1]	42 (29/13/0)	Peru [2]	493 (283/199/11)	Peru [3]	751 (501/250/0)	Peru 1 and 2 [5]	5	Quechua_Coriell [6]
Philippines (PHIL)	9.8	125.5	162 (124/38/0)	Philippines [1]	41 (30/11/0)	Philippines [2]	292 (189/103/0)	Philippines [3]	58 (39/19/0)	Philippines no 2 Calatagan BP [4]	21	Visayan, Kankanaey, Ilocano, Tagalog [8]
Somalia (SOMA)	5.6	48.3	66 (63/3/0)	Somalia [1]	34 (34/0/0)	Somalia [2]	83 (67/12/4)	Somalia [3]	77 (51/26/0)	Somalia [4]	13	Somali [6]
South Africa (SAFR)	-27.8	21.1	56 (50/6/0)	South Africa Khoi-san [1]	9 (8/1/0)	South Africa Khoi-san [2]	73 (48/19/6)	South Africa Khoi-san [3]	167 (111/56/0)	San plus Riet River (San; >12-19th Cent) [4]	16	Khomani, Ju_hoan_North [6,10]
South Finland (SFIN)	60.2	24.9	25 (25/0/0)	Finland [1]	5 (5/0/0)	Finland [2]	26 (26/0/0)	Finland [3]	51 (34/17/0)	Ladoga Finns [4]	56	Finland_POPRES, Finnish_FIN, Finland [6,9,14]
Thailand (THAI)	13.8	100.5	58 (47/11/0)	Thailand [1]	20 (18/2/0)	Thailand [2]	77 (49/28/0)	Thailand [3]	189 (126/63/0)	Recent Thailand [4]	10	Thai [6]
Vanuatu (VANU)	-16.5	167.24	80 (51/29/0)	New Hebrides [1]	48 (33/15/0)	New Hebrides [2]	204 (129/75/0)	New Hebrides [3]	78 (52/26/0)	New Hebrides [5]	176	Vanuatu [15]
Vietnam (VIET)	21	105.9	24 (24/0/0)	Vietnam [1]	6 (6/0/0)	Vietnam [2]	36 (25/11/0)	Vietnam [3]	76 (51/25/0)	Recent Tonkin, Historic Annam [4]	18	Kinh_Vietnam_KHV, Vietnamese [6,8]
Total			2994 (2137/857/0)		909 (717/192/0)		4623 (2718/1791/114)		3470 (2315/1155/0)		857	

[1] refs. (23) and (24)
[2] ref. (25)
[3] ref. (26)
[4] ref. (27)
[5] ref. (2)
[6] ref. (28)
[7] ref. (29)
[8] ref. (30)
[9] ref. (31)

[10] ref. (32)
[11] ref. (33)
[12] ref. (34)
[13] ref. (35)
[14] ref. (36)
[15] ref. (37)

Table S2. Correlations between phenotypic (D^2), genetic (F_{ST}), climate (C), and geographic (G) distances.

	D^2 - F_{ST}	D^2 - C	D^2 - G	D^2 - F_{ST}, C	D^2 - F_{ST}, G
Cranial metric	0.5525	0.4082	0.3638	0.6180	0.4466
Cranial non-metric	0.3792	0.1679	0.4050	0.3895	0.1555
Dental metric	0.2146	0.1750	0.1490	0.2230	0.1562
Dental non-metric	0.5887	0.0615	0.4792	0.5917	0.4088
Craniodental combined	0.6393	0.3177	0.4742	0.6839	0.4911

Reported values are Pearson correlation coefficients (r); D^2: phenotypic between-population differentiation distances; F_{ST}: genetic between-population distances; C: climate between-population distances; G: geodesic between-population distances. The correlation between F_{ST} and C is r=-0.0280. The correlation between F_{ST} and G is r=0.6781. The correlation between C and G is r=0.0647.

Table S3. Repeated-measures *t*-tests among pairs of neutrality estimate distributions for five craniodental data types, correcting for population sampling bias.

	Cranial metric	Cranial non-metric	Dental metric	Dental non-metric	Craniodental combined
Cranial metric	-	224.1573	281.3423	23.8932	-59.3443
Cranial non-metric	**<0.000001**	-	127.0718	-201.2013	-293.7984
Dental metric	**<0.000001**	**<0.000001**	-	-263.7431	-340.6830
Dental non-metric	**<0.000001**	**<0.000001**	**<0.000001**	-	-83.7165
Craniodental combined	**<0.000001**	**<0.000001**	**<0.000001**	**<0.000001**	-

Reported values are *t* values (above the diagonal) and two-tailed *p* values (below the diagonal). Degrees of freedom are 999 in all cases. Bold type indicates significance after Bonferroni correction (α=0.005).

Table S4. Repeated-measures *t*-tests among pairs of neutrality estimate distributions for five craniodental data types, correcting for population and phenotype sampling bias.

	Cranial metric	Cranial non-metric	Dental metric	Dental non-metric	Craniodental combined
Cranial metric	-	146.6507	183.5531	-10.1956	-45.6016
Cranial non-metric	**<0.000001**	-	102.7687	-182.5141	-194.1206
Dental metric	**<0.000001**	**<0.000001**	-	-200.1497	-222.6323
Dental non-metric	**<0.000001**	**<0.000001**	**<0.000001**	-	-38.8352
Craniodental Combined	**<0.000001**	**<0.000001**	**<0.000001**	**<0.000001**	-

Reported values are *t* values (above the diagonal) and two-tailed *p* values (below the diagonal). Degrees of freedom are 999 in all cases. Bold type indicates significance after Bonferroni correction (α=0.005).

Table S5. Repeated-measures *t*-tests among pairs of neutrality estimate distributions for five craniodental data types, correcting for population, phenotype, and loci sampling bias.

	Cranial metric	Cranial non-metric	Dental metric	Dental non-metric	Craniodental combined
Cranial metric	-	38.8674	73.0812	-3.8637	-16.8133
Cranial non-metric	**<0.000001**	-	37.9779	-39.9409	-58.2535
Dental metric	**<0.000001**	**<0.000001**	-	-73.5707	-90.3837
Dental non-metric	**0.000119**	**<0.000001**	**<0.000001**	-	-11.7211
Craniodental combined	**<0.000001**	**<0.000001**	**<0.000001**	**<0.000001**	-

Reported values are *t* values (above the diagonal) and two-tailed *p* values (below the diagonal). Degrees of freedom are 999 in all cases. Bold type indicates significance after Bonferroni correction (α=0.005).

Table S6. Neutrality estimates for five craniodental data types using males only and utilizing size-corrected metric data, calculated as partial Pearson correlation (*r*) between phenotypic (D^2) and neutral genetic (F_{ST}) distances across 26 modern human population samples, controlling for climate distances.

Craniodental data type	Population sampling bias correction[1]	Population and phenotype sampling bias correction[2]	Population, phenotype, and loci sampling bias correction[3]
Cranial metric	0.591 (0.560 – 0.666)	0.557 (0.487 – 0.635)	0.444 (0.261 – 0.604)
Cranial non-metric	0.340 (0.299 – 0.411)	0.340 (0.299 – 0.411)	0.270 (0.105 – 0.447)
Dental metric	0.234 (0.167 – 0.301)	0.229 (0.139 – 0.316)	0.183 (0.003 – 0.395)
Dental non-metric	0.556 (0.498 – 0.599)	0.553 (0.487 – 0.607)	0.446 (0.233 – 0.640)
Craniodental combined	0.651 (0.611 – 0.720)	0.623 (0.548 – 0.696)	0.502 (0.333 – 0.656)

[1] Median (and 95% range) of 1,000 iteratively generated *r* values, each iteration leaving out a randomly selected population in the phenotypic and genomic datasets and a randomly selected individual in each remaining population.
[2] Median (and 95% range) of 1,000 iteratively generated *r* values, each iteration randomly undersampling the number of phenotypic variables, combined with population sampling bias correction.
[3] Median (and 95% range) of 1,000 iteratively generated *r* values, each iteration randomly undersampling the number of loci, combined with population and phenotype sampling bias correction.

Table S7. Neutrality estimates for five craniodental data types using males only and without size-correction applied to the metric data, calculated as partial Pearson correlation (*r*) between phenotypic (D^2) and neutral genetic (F_{ST}) distances across 26 modern human population samples, controlling for climate distances.

Craniodental data type	Population sampling bias correction[1]	Population and phenotype sampling bias correction[2]	Population, phenotype, and loci sampling bias correction[3]
Cranial metric	0.557 (0.526 – 0.644)	0.518 (0.448 – 0.599)	0.420 (0.223 – 0.579)
Cranial non-metric	0.340 (0.299 – 0.410)	0.340 (0.299 – 0.410)	0.276 (0.117 – 0.451)
Dental metric	0.290 (0.223 – 0.352)	0.288 (0.193 – 0.368)	0.222 (0.013 – 0.438)
Dental non-metric	0.558 (0.501 – 0.601)	0.556 (0.486 – 0.607)	0.447 (0.228 – 0.633)
Craniodental combined	0.638 (0.599 – 0.722)	0.615 (0.538 – 0.699)	0.491 (0.333 – 0.641)

[1] Median (and 95% range) of 1,000 iteratively generated *r* values, each iteration leaving out a randomly selected population in the phenotypic and genomic datasets and a randomly selected individual in each remaining population.

[2] Median (and 95% range) of 1,000 iteratively generated *r* values, each iteration randomly undersampling the number of phenotypic variables, combined with population sampling bias correction.

[3] Median (and 95% range) of 1,000 iteratively generated *r* values, each iteration randomly undersampling the number of loci, combined with population and phenotype sampling bias correction.

Table S8. Neutrality estimates for five craniodental data types, calculated as partial Pearson correlation (r) between phenotypic (D^2) and neutral genetic (F_{ST}) distances across a subset of 16 modern human populations with samples sizes n≥10, controlling for climate distances.

Craniodental data type	Population sampling bias correction[1]	Population and phenotype sampling bias correction[2]	Population, phenotype, and loci sampling bias correction[3]
Cranial metric	0.644 (0.620 – 0.715)	0.585 (0.498 – 0.686)	0.489 (0.301 – 0.662)
Cranial non-metric	0.516 (0.463 – 0.604)	0.516 (0.463 – 0.604)	0.441 (0.236 – 0.603)
Dental metric	0.456 (0.326 – 0.547)	0.450 (0.297 – 0.539)	0.369 (0.067 – 0.612)
Dental non-metric	0.550 (0.484 – 0.608)	0.543 (0.473 – 0.613)	0.462 (0.228 – 0.701)
Craniodental combined	0.767 (0.743 – 0.820)	0.727 (0.658 – 0.790)	0.617 (0.418 – 0.764)

[1] Median (and 95% range) of 1,000 iteratively generated r values, each iteration leaving out a randomly selected population in the phenotypic and genomic datasets and a randomly selected individual in each remaining population.

[2] Median (and 95% range) of 1,000 iteratively generated r values, each iteration randomly undersampling the number of phenotypic variables, combined with population sampling bias correction.

[3] Median (and 95% range) of 1,000 iteratively generated r values, each iteration randomly undersampling the number of loci, combined with population and phenotype sampling bias correction.

Table S9. Estimates of effective population size (N_e) calculated from levels of linkage disequilibrium.

Population	n	N_e
ALEU	7	2229 (1498 – 3419)
ARIZ	14	1345 (911 – 2361)
AUST	10	2863 (1189 – 4904)
BORN	17	3498 (2646 – 5400)
ENGL	24	4915 (2952 – 6620)
ESIB	23	2040 (1556 – 2877)
GREE	43	6493 (4519 – 8218)
ITAL	33	5679 (3985 – 7839)
JAPA	29	3916 (2803 – 4929)
KENY	4	1968 (1187 – 3746)
MALA	9	2774 (1685 – 3943)
MONG	10	3187 (1679 – 4457)
NBRI	156	3631 (2172 – 5082)
NEPA	10	2196 (1678 – 4043)
NETH	16	3798 (2434 – 5251)
NFEN	3	3820 (1091 – Inf)
NGUI	49	3395 (1382 – 4615)
NIGE	85	9832 (8542 – 15632)
PERU	5	2296 (1009 – 6383)
PHIL	21	3534 (2721 – 4821)
SAFR	16	4386 (3930 – 6382)
SFIN	56	4803 (3324 – 6350)
SOMA	13	4718 (2837 – 8451)
THAI	10	2690 (1542 – 3713)
VANU	176	4390 (3096 – 8320)
VIET	18	4124 (3022 – 5676)

Reported N_e values are median (and 95% range).

Table S10. Neutrality estimates for five craniodental data types, calculated as partial Pearson correlation (*r*) between phenotypic (P_{ST}) and neutral genetic (F_{ST}) distances across 26 modern human population samples, controlling for climate distances.

Craniodental data type	Population sampling bias correction[1]	Population and phenotype sampling bias correction[2]	Population, phenotype, and loci sampling bias correction[3]
Cranial metric	0.556 (0.498 – 0.633)	0.538 (0.474 – 0.613)	0.433 (0.241 – 0.604)
Cranial non-metric	0.253 (0.210 – 0.314)	0.253 (0.210 – 0.314)	0.208 (0.030 – 0.385)
Dental metric	0.134 (0.062 – 0.235)	0.136 (0.022 – 0.235)	0.101 (-0.065 – 0.301)
Dental non-metric	0.524 (0.441 – 0.569)	0.519 (0.439 – 0.569)	0.422 (0.198 – 0.607)
Craniodental combined	0.514 (0.449 – 0.582)	0.491 (0.389 – 0.584)	0.402 (0.188 – 0.587)

[1] Median (and 95% range) of 1,000 iteratively generated *r* values, each iteration leaving out a randomly selected population in the phenotypic and genomic datasets and a randomly selected individual in each remaining population.

[2] Median (and 95% range) of 1,000 iteratively generated *r* values, each iteration randomly undersampling the number of phenotypic variables, combined with population sampling bias correction.

[3] Median (and 95% range) of 1,000 iteratively generated *r* values, each iteration randomly undersampling the number of loci, combined with population and phenotype sampling bias correction.

Legends for Datafiles S1 to S13

Dataset S1 (separate Microsoft Excel file). F_{ST} distance matrix.

Dataset S2 (separate Microsoft Excel file). Cranial metric D^2 distance matrix.

Dataset S3 (separate Microsoft Excel file). Dental metric D^2 distance matrix.

Dataset S4 (separate Microsoft Excel file). Cranial non-metric D^2 distance matrix.

Dataset S5 (separate Microsoft Excel file). Dental non-metric D^2 distance matrix.

Dataset S6 (separate Microsoft Excel file). Craniodental metric and non-metric combined D^2 distance matrix.

Dataset S7 (separate Microsoft Excel file). Climate *C* distance matrix.

Dataset S8 (separate Microsoft Excel file). Geographic *G* distance matrix.

Dataset S9 (separate Microsoft Excel file). Summary statistics of the *k*NN-imputed and size-corrected cranial metric dataset.

Dataset S10 (separate Microsoft Excel file). Summary statistics of the *k*NN-imputed and size-corrected dental metric dataset.

Dataset S11 (separate Microsoft Excel file). Summary statistics of the cranial non-metric trait dataset.

Dataset S12 (separate Microsoft Excel file). Summary statistics of the dental non-metric trait dataset.

Dataset S13 (separate Microsoft Excel file). Climate variables for the matched population samples.

SI References

1. G. R. Scott, C. G. Turner II, G. C. Townsend, M. Martinón-Torres, *The Anthropology of Modern Human Teeth* (Cambridge University Press, 2018).
2. G. R. Scott, J. D. Irish, *Human Tooth Crown and Root Morphology* (Cambridge University Press, 2017).
3. H. Rathmann, H. Reyes-Centeno, Testing the utility of dental morphological trait combinations for inferring human neutral genetic variation. *Proc. Natl. Acad. Sci. U.S.A.* **117**, 10769–10777 (2020).
4. G. R. Scott et al., Peopling the Americas: Not “Out of Japan”. *PaleoAmerica* **7**, 309–332 (2021).
5. C. M. Stojanowski, K. S. Paul, A. C. Seidel, W. N. Duncan, D. Guatelli-Steinberg, Heritability and genetic integration of anterior tooth crown variants in the South Carolina Gullah. *Am. J. Phys. Anthropol.* **167**, 124–143 (2018).
6. K. S. Paul, C. M. Stojanowski, T. E. Hughes, A. H. Brook, G. C. Townsend, Patterns of heritability across the human diphyodont dental complex: Crown morphology of Australian twins and families. *Am. J. Phys. Anthropol.* **172**, 447–461 (2020).
7. L. W. Konigsberg, "A post-Neumann history of biological and genetic distance studies in bioarchaeology" in *Bioarchaeology: The contextual analysis of human remains*, J. E. Buikstra, L. A. Beck, Eds. (Academic Press, 2006), pp. 263–279.
8. J. H. Relethford, "Biological distances and population genetics in bioarchaeology" in *Biological distance analysis: Forensic and bioarchaeological perspectives*, M. A. Pilloud, J. T. Hefner, Eds. (Academic Press, 2016), pp. 23–33.
9. H. Reyes-Centeno, S. Ghirotto, K. Harvati, Genomic validation of the differential preservation of population history in modern human cranial anatomy. *Am. J. Phys. Anthropol.* **162**, 170–179 (2017).
10. H. Reyes-Centeno, K. Harvati, G. Jager, Tracking modern human population history from linguistic and cranial phenotype. *Sci. Rep.* **6**, 36645 (2016).
11. H. Rathmann et al., Reconstructing human population history from dental phenotypes. *Sci. Rep.* **7**, 12495 (2017).
12. H. Harpending, R. Ward, "Chemical systematics and human populations" in *Biochemical aspects of evolutionary biology*, M. H. Nitecki, Ed. (University of Chicago Press, 1982), pp. 213–256.
13. J. H. Relethford, M. H. Crawford, J. Blangero, Genetic drift and gene flow in post-famine Ireland. *Hum. Biol.* **69**, 443–465 (1997).
14. C. M. Stojanowski, K. S. Paul, A. C. Seidel, W. N. Duncan, D. Guatelli-Steinberg, Heritability and genetic integration of tooth size in the South Carolina Gullah. *Am. J. Phys. Anthropol.* **164**, 505–521 (2017).
15. C. M. Stojanowski, K. S. Paul, A. C. Seidel, W. N. Duncan, D. Guatelli-Steinberg, Quantitative genetic analyses of postcanine morphological crown variation. *Am. J. Phys. Anthropol.* **168**, 606–631 (2019).
16. N. Martínez-Abadías et al., Heritability of human cranial dimensions: comparing the evolvability of different cranial regions. *J. Anat.* **214**, 19–35 (2009).
17. J. M. Cheverud, J. E. Buikstra, Quantitative genetics of skeletal nonmetric traits in the rhesus macaques on Cayo Santiago. II. Phenotypic, genetic, and environmental correlations between traits. *Am. J. Phys. Anthropol.* **54**, 51–58 (1981).
18. E. A. Carson, Maximum-likelihood variance components analysis of heritabilities of cranial nonmetric traits. *Hum. Biol.* **78**, 383–402 (2006).

19. L. J. Hlusko, R. D. Sage, M. C. Mahaney, Modularity in the mammalian dentition: mice and monkeys share a common dental genetic architecture. *J. Exp. Zool. B. Mol. Dev. Evol.* **316**, 21–49 (2011).
20. E. A. Carson, Maximum likelihood estimation of human craniometric heritabilities. *Am. J. Phys. Anthropol.* **131**, 169–180 (2006).
21. M. Mezzavilla, Neon: An R Package to Estimate Human Effective Population Size and Divergence Time from Patterns of Linkage Disequilibrium between SNPS. *J Comput Sci Syst Biol* **8 (**2015).
22. F. Tassi et al., Early modern human dispersal from Africa: genomic evidence for multiple waves of migration. *Investig. Genet.* **6**, 13 (2015).
23. T. Hanihara, Comparison of craniofacial features of major human groups. *Am. J. Phys. Anthropol.* **99**, 389–412 (1996).
24. M. Hubbe, T. Hanihara, K. Harvati, Climate signatures in the morphological differentiation of worldwide modern human populations. *Anat. Rec.* **292**, 1720–1733 (2009).
25. T. Hanihara, H. Ishida, Metric dental variation of major human populations. *Am. J. Phys. Anthropol.* **128**, 287–298 (2005).
26. T. Hanihara, H. Ishida, Y. Dodo, Characterization of biological diversity through analysis of discrete cranial traits. *Am. J. Phys. Anthropol.* **121**, 241–251 (2003).
27. J. D. Irish, A. Morez, L. Girdland Flink, E. L. W. Phillips, G. R. Scott, Do dental nonmetric traits actually work as proxies for neutral genomic data? Some answers from continental- and global-level analyses. *Am. J. Phys. Anthropol.* **172**, 347–375 (2020).
28. I. Lazaridis et al., Ancient human genomes suggest three ancestral populations for present-day Europeans. *Nature* **513**, 409–413 (2014).
29. P. Qin, M. Stoneking, Denisovan ancestry in east Eurasian and native American populations. *Mol. Biol. Evol.* **32**, 2665–2674 (2015).
30. P. Skoglund et al., Genomic insights into the peopling of the Southwest Pacific. *Nature* **538**, 510–513 (2016).
31. M. R. Nelson et al., The Population Reference Sample, POPRES: a resource for population, disease, and pharmacological genetics research. *Am. J. Hum. Genet.* **83**, 347–358 (2008).
32. J. K. Pickrell, J. K. Pritchard, Inference of population splits and mixtures from genome-wide allele frequency data. *PLoS Genet.* **8**, e1002967 (2012).
33. A. Bergström et al., Insights into human genetic variation and population history from 929 diverse genomes. *Science* **367 (**2020).
34. B. M. Henn et al., Genomic ancestry of North Africans supports back-to-Africa migrations. *PLoS Genet.* **8**, e1002397 (2012).
35. S. Mallick et al., The Simons Genome Diversity Project: 300 genomes from 142 diverse populations. *Nature* **538**, 201–206 (2016).
36. O. Lao et al., Correlation between genetic and geographic structure in Europe. *Curr. Biol.* **18**, 1241–1248 (2008).
37. M. Lipson et al., Population Turnover in Remote Oceania Shortly after Initial Settlement. *Curr. Biol.* **28**, 1157-1165.e7 (2018).
